# A computational study of the thortveitite structure of zinc pyrovanadate, Zn_2_V_2_O_7_, under pressure

**DOI:** 10.1039/d3ra02426a

**Published:** 2023-06-08

**Authors:** S. Reza, M. Maaza, M. S. Islam

**Affiliations:** a Department of Physics, University of Rajshahi Rajshahi 6205 Bangladesh sislamru@gmail.com; b UNESCO-UNISA Africa Chair in Nanosciences-Nanotechnology, College of Graduate Studies, University of South Africa Muckleneuk Ridge, PO Box 392 Pretoria South Africa; c Nanosciences African Network (NANOAFNET), Materials Research Dept., iThemba LABS-National Research Foundation of South Africa 1 Old Faure Road, Somerset West, PO Box 722 Western Cape 7129 South Africa

## Abstract

We performed a pressure-driven study of zinc pyrovanadate, Zn_2_V_2_O_7_, using the first-principles approach under the framework of density functional theory (DFT). Zn_2_V_2_O_7_ crystalizes in a monoclinic (α-phase) structure with the space group *C*2/*c* at ambient pressure. In comparison with the ambient phase, there are four different high-pressure phases, namely β, γ, κ and δ, found at 0.7, 3.8, 4.8 and 5.3 GPa, respectively. The detailed crystallographic analysis as well as their structures is consistent with the theory and experiment reported in the literature. All phases including the ambient phase are mechanically stable, elastically anisotropic and malleable. The compressibility of the studied pyrovanadate is higher than that of the other *meta*- and pyrovanadates. The energy dispersion of these studied phases reveals that they are indirect band gap semiconductors with wide band gap energies. The band gap energies follow a reduced trend with pressure except the κ-phase. The effective masses for all of these studied phases were computed from their corresponding band structures. The values of energy gaps obtained from the band structures are almost similar to the optical band gap obtained from the optical absorption spectra, as estimated by the Wood–Tauc theory.

## Abstract 2

The ambient (α) phase of Zn_2_V_2_O_7_ shows polymorphic structures that are pressure dependent. All polymorphs exhibit stability, anisotropy, malleability, and indirect band gap semiconductivity.

## Graphical abstract

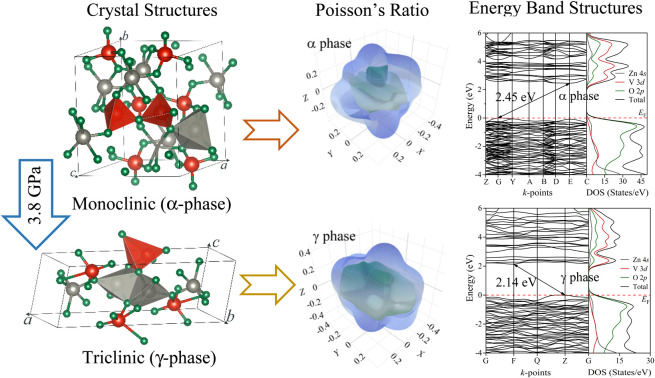

## Introduction

The search for chemical systems suitable for lithium-ion batteries with good charge-exchange ability and high energy density^1–3^ has attracted the attention of researchers toward electrode materials to meet the energy demand in portable electronic devices such as cell phones, laptops, computers, digital cameras, and motor vehicles due to their storage capacity which commercial batteries made of graphite lack. In this context, vanadium-based electrode materials, such as metavanadates (MV_2_O_6_), pyrovanadates (M_2_V_2_O_7_) and orthovanadates (M_3_V_2_O_8_) (M = divalent cations), have been widely used for many technological applications as electrodes in lithium-ion rechargeable batteries and supercapacitors, as long-life cathode materials of aqueous zinc-ion batteries and as phosphors for white light-emitting diodes.^4–9^ Zinc pyrovanadate, Zn_2_V_2_O_7_, can be a better candidate than metavanadates and orthovanadates for use as a hydrogen storage material, as it shows a high storage capacity of more than 2899 mA h g^−1^ after 20 cycles.^10^ The latest experiment on nickel pyrovanadate reported excellent electrochemical properties with a high stable specific capacity of ∼960 mA h g^−1^ at a current density of 100 mA g^−1^.^11^

Tremendous attention has recently been paid to study Zn_2_V_2_O_7_, isostructural with thortveitite mineral Sc_2_Si_2_O_7_,^12^ due to its above-mentioned fascinating behavior. First, Makarov *et al.* experimentally crystallized zinc pyrovanadate and observed two polymorphic structures within the transition temperature range of 608–620 °C.^13^ The low-temperature phase of α-Zn_2_V_2_O_7_ immediately crystallizes in a monoclinic system with the space group (SG) of *C*2/*c*.^14^ The other polymorph of Zn_2_V_2_O_7_, high-temperature β-phase, forms a thortveitite structure in the same crystal with the SG of *C*2/*m*.^15^ Besides structural transitions upon temperature stimulation, the behavior of Zn_2_V_2_O_7_ is interesting under high-pressure conditions. A much recent report on Zn_2_V_2_O_7_ based on XRD patterns at a pressure up to 12 GPa has surprisingly indicated at least three structural phase transitions but no pressure-driven transitions have been shown in metavanadate ZnV_2_O_6_ and orthovanadate Zn_3_V_2_O_8_.^16^ Interestingly, the studied pyrovanadate under ambient conditions is highly compressible in comparison with the *meta*- and *ortho*-vanadates. The first structural transition was found at 0.7 GPa, where the ambient phase of α-polymorph was transformed into β-polymorph although the structure was monoclinic.^14,16^ The second high-pressure phase transition of the γ-phase appeared at 3.8 GPa, and the proposed structure was triclinic with SG *P*1̄. The third phase transition, namely, the post γ-phase appeared at 10.8 GPa, but the structure was unidentified.^16^

Very recently, first-principles calculations based on density functional theory (DFT) revealed several new additional phases of κ-Zn_2_V_2_O_7_ isomorphic to Ni_2_V_2_O_7_ (SG *P*2_1_/*c*),^17^ δ-Zn_2_V_2_O_7_ structure corresponding to Hg_2_V_2_O_7_ (SG *Pnma*),^18^ ε-Zn_2_V_2_O_7_ similar to Pb_2_V_2_O_7_ (SG *P*2_1_/*c*),^19^ and ω-Zn_2_V_2_O_7_ corresponding to Sr_2_V_2_O_7_ (SG *P*1̄),^20^ in association with α-, β- and γ-phases on the basis of crystal-chemistry arguments.^21,22^ In this work, they predicted that the post γ-phase could be the κ-phase, although the structures are largely different. This comparison to define the high-pressure phase is still a subject of debate. The present work used first-principles DFT-encoded CASTEP to determine the post γ-phase in connection with the above-mentioned phases except ε- and ω-phases. In association with energy dispersion and optical absorption, the purpose is to show mechanical stability under compression by studying the elastic behavior of all these phases. In this work, the behavior of this pyrovanadate and their phase-related discussion will be carried out under pressure.

## Theoretical details

The computational work was carried out by optimizing the geometry *via* minimization of the total energy using the CAmbridge Serial Total Energy Package (CASTEP) code^23^ based upon density functional theory (DFT). Generalized gradient approximation (GGA) of Perdew–Burke–Ernzerhof (PBE)^24^ was the exchange–correlation functional. To compute the other polymorphs compared with the ambient phase of the studied pyrovanadate, the different hydrostatic pressure on the structures was applied before optimizing the geometry as reported in the experiment as well as theoretical prediction given by Díaz-Anichtchenko *et al.*^16,21^ The plane wave energy cut-off 600 eV was used for all these studied pyrovanadate. Monkhorst–Pack^25^ grid parameters 6 × 5 × 5 were set for the sampling of the Brillouin zone in almost all of these studied phases except the δ-phase (6 × 11 × 2). The set of OTFG ultrasoft pseudopotentials were used in the calculations for describing the electron–ion interactions.^26^ After getting the optimized geometries for these studied phases, we successively calculated different properties such as elastic, electronic and optical, which are discussed in the next section. Finally, ELATE, an open-source Python module for the analysis of elastic tensors, was used to visualize the 3D image of elastic moduli for describing the nature of anisotropy.^27^

## Results and discussion

**Fig. 1:**
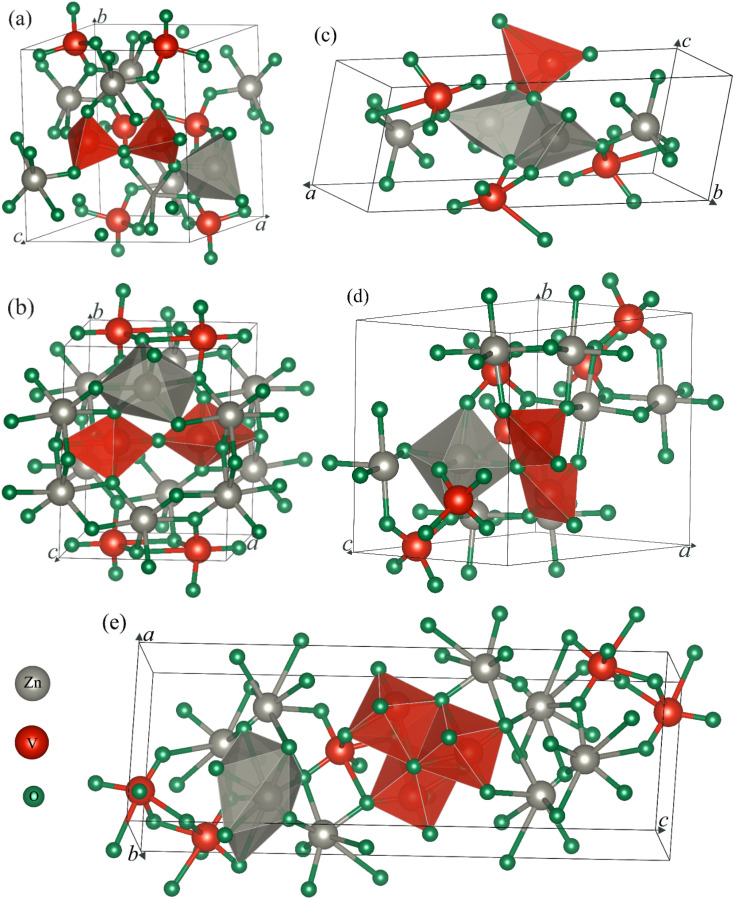
Crystal structures of the different (a) α-, (b) β-, (c) γ-, (d) κ- and (e) δ-phases of zinc pyrovanadate, Zn_2_V_2_O_7_. Here, the gray, red and green atoms in these crystals are defined as Zn, V and O, respectively.

First, we draw the crystal structure of an ambient phase of α-Zn_2_V_2_O_7_, which is sketched in Fig. 1a. As shown in Fig. 1a, α-Zn_2_V_2_O_7_ crystallizes in the monoclinic system with the space group (SG) of *C*2/*c* (no. 15). The unit cell of α-Zn_2_V_2_O_7_ contains 44 atoms. The optimized lattice parameters are presented in Table 1, which are almost consistent with the available experimental and theoretical results.^14–16,21^ In addition, the first high-pressure phase of β-Zn_2_V_2_O_7_ forms a thortveitite structure, as illustrated in Fig. 1b, under the same crystal with the SG of *C*2/*m*, which is similar to the observations reported in the literature.^15,16,21^ Recently, pressure-driven structural transitions have been found in Zn_2_V_2_O_7_ instead of metavanadate ZnV_2_O_6_ and orthovanadate Zn_3_V_2_O_8_.^16^ Within the pressure range of 10^−4^ to 14.7 GPa, the first polymorph appeared at 0.7 GPa, which is isostructural with Cd_2_V_2_O_7_.^28^ However, the second high-pressure phase corresponding to the γ-phase at 3.8 GPa was mentioned with a triclinic structure (*P*1̄), isomorphic to Mg_2_V_2_O_7_,^29^ as shown in Fig. 1c. Several new additional phases of the κ-Zn_2_V_2_O_7_ structure corresponding to Ni_2_V_2_O_7_ (*P*2_1_/*c*)^17^ and δ-Zn_2_V_2_O_7_ crystals isomorphic to Hg_2_V_2_O_7_ (*Pnma*)^18^ have recently been proposed on the basis of their crystal-chemistry arguments.^21,22^ These predicted phases are subsequently found at 4.8 and 5.2 GPa, respectively, and their corresponding structures are demonstrated in Fig. 1d and e. According to the XRD pattern given in Díaz-Anichtchenko *et al.* work,^16^ they have found an unidentified structure at 10.8 GPa, namely, the post γ-phase, that is probably relevant to the κ-phase.^17^ Our comment, in this regard, on the basis of the present computations will be mentioned later.

**Table 1:** Crystallographic information, number of formula units in the unit cell (*Z*), optimized lattice parameters and average atomic bond distances in Zn and V polyhedra for all of these studied phases of Zn_2_V_2_O_7_

Phases	Crystal system	Space group	*Z*	Lattice parameters				Bond length (Å)		Ref.
				Axial length (Å)			Axial angle (°)			
				*a*	*b*	*c*		Zn–O	V–O	
α	Monoclinic	*C*2/*c*	4	7.455	8.380	10.254	*α* = *γ* = 90	2.157	1.789	This work
				7.429	8.340	10.098 (ref. 14)	*β* = 111.069			
β		*C*2/*m*	2	6.975	8.503	5.046	*α* = *γ* = 90	2.137	1.866	
				6.932	8.440	5.033 (ref. 15)	*β* = 108.846			
γ	Triclinic	*P*1̄	2	13.636	5.444	5.108	*α* = 77.916	2.059	1.906	
				13.621	5.235	4.923 (ref. 16)	*β* = 107.948			
							*γ* = 130.960			
κ	Monoclinic	*P*2_1_/*c*	4	6.651	8.324	9.450	*α* = *γ* = 90	2.113	1.722	
				6.615	8.394	9.492 (ref. 21)	*β* = 100.133			
δ	Orthorhombic	*Pnma*	4	6.840	3.538	19.530	*α* = *β* = *γ* = 90	2.323	1.926	
				6.865	3.578	19.601 (ref. 21)				

As illustrated in Fig. 1a, the cations (Zn) in the ambient phase occupy sites with the nearest neighbors of five oxygen (O) atoms as compared with the cations in the thortveitite-like structure coordinated to be six-fold.^14,16^ The average distances of both of these polyhedral made of cations and anions are about 2 Å. However, the vanadium (V) atoms are tetrahedrally coordinated. Each pair of tetrahedra [VO_4_], slightly distorted, is connected by a common O atom located at a common corner to form the [V_2_O_7_]^4−^ pyrovanadate anions present in the thortveitite-like minerals.^30–32^ The two cations per formula unit in this structure donate four electrons to the [V_2_O_7_] group, which is converted into [V_2_O_7_]^4−^ anions based on the concept given by Zintl-Klemm.^30^ A similar structural pattern is seen in the first transition phase of Zn_2_V_2_O_7_ except the coordination of cations and anions, as shown in Fig. 1b. The Zn atoms in the β-phase form a polyhedra, ZnO_6_, but the vanadium is coordinated with five O atoms. In the case of γ-phase, the two cations, each being five-coordinated, are connected by two common oxygen atoms, while a single oxygen atom is common between the anionic VO_5_ and the two cationic polyhedra, as depicted in Fig. 1c. The κ-phase in Fig. 1d is structurally consistent with the thortveitite-like structure, where Zn and V atoms are bonded with six and four O atoms, respectively. Here, one oxygen atom connects two tetrahedra of V and a polyhedron of Zn. Higher coordination of cations (octahedra) and anions (hexahedra) is formed in the δ-phase, as shown in Fig. 1e. The three hexahedra in this structure are linked by common oxygen atoms.

In order to study the mechanical stability of a material, the mechanical behavior of a crystal lattice can be expressed by its matrix of second-order elastic/stiffness constants:1
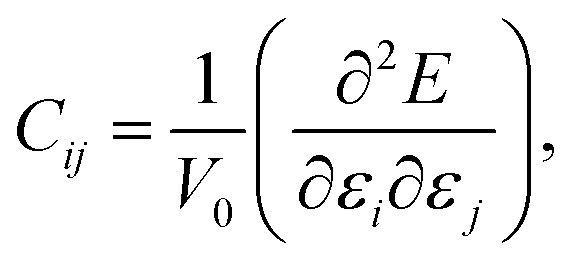
where *E* is the energy of the crystal, *V*_0_ is its equilibrium volume and *ε* denotes a strain.^33^ The size of stiffness matrix is 6 × 6, which has 21 nonzero independent components. Out of these studied phases of Zn_2_V_2_O_7_, the monoclinic structures of α, β and κ phases, orthorhombic structure of δ phase and triclinic structure of γ phase have 13, 9 and 21 stiffness constants, respectively.^33^ The values of elastic constants calculated from the first-principles DFT for the five different phases of Zn_2_V_2_O_7_ are presented in Table 2.

**Table 2:** Stiffness constants *C*_*ij*_ (GPa) for different phases of Zn_2_V_2_O_7_

→	*C*_11_	*C*_12_	*C*_13_	*C*_15_	*C*_22_	*C*_23_	*C*_25_	*C*_33_	*C*_35_	*C*_44_	*C*_46_	*C*_55_	*C*_66_
Phases													
α	135	61	61	5	145	64	7	120	11	44	8	50	32
β	356	41	91	23	184	105	−10	186	9	30	−29	38	−15
κ	169	72	72	17	221	100	1	156	8	56	3	40	55
δ	181	109	81	—	275	90	—	201	—	34	—	53	67
γ	162	87	79	7	161	91	5	161	4	41	2	42	37
→	*C*_14_	*C*_16_	*C*_24_	*C*_26_	*C*_34_	*C*_36_	*C*_45_	*C*_56_					
γ	−0.5	−12	−3	−6	−12	−10	−6	4					

Depending on the stiffness constants, we determine the mechanical stability of these studied phases of Zn_2_V_2_O_7_ using the well-known Born–Huang criteria.^34^ All these phases are mechanically stable except β, which has negative values for *C*_66_. However, the majority of the criteria based on stiffness values in the β-phase are fulfilled. It is noteworthy to mention that the stability of γ is unaffected by modest negative values of some elastic constants. This would rather suggest that the crystal in these phases may have minor internal stresses.^35^

As a polycrystalline mechanical property, we also estimated the modulus of elasticity, *e.g.* the bulk modulus *B*_H_ = (*B*_V_ + *B*_R_)/2, where *B*_V_ is Voigt's bulk modulus and *B*_R_ is Reuss's bulk modulus; and the shear modulus *G*_H_ = (*G*_V_ + *G*_R_)/2, where *G*_V_ is Voigt's shear modulus and *G*_R_ is Reuss's shear modulus using the average approximations given by Hill.^36^ Young's modulus (*E*) and Poisson's ratio (*ν*) were also computed using the relationships: *E* = 9*B*_H_*G*_H_/(3*B*_H_ + *G*_H_) and *ν* = (3*B*_H_ − *E*)/(6*B*_H_), respectively.^37^ The calculated values of *B*_H_, *G*_H_, *E* and *ν*, were compared with the previously reported DFT results, which are listed in Table 3. It is well known that the value of bulk modulus describes the compressibility of a material. In this context, β, γ, κ and δ phases are the least compressible in comparison with the α phase.^21^ Moreover, the compressibility can be enhanced by increasing the unit cell volume because of an inverse relationship between the bulk modulus and the unit cell volume under ambient conditions.^21,38^ The calculated values of bulk modulus were higher than the result published in the literature.^21,38^ Again the value of either bulk modulus or shear modulus can indirectly measure the hardness of a material. Using Hill's approximation, the shear modulus is much smaller than the bulk modulus, reflecting that the shear deformation is easier as shown in Table 3.^36^ Generally, the stiffness of an elastic material can be identified by the value of Young's modulus, so that the β phase is, in this case, highly stiffer than the others. The value of Pugh's ratio (*B*/*G*) distinguishes the ductile (>1.74) and brittle (<1.74) nature of materials.^39^ As listed in Table 3, the most malleable behavior is expected in these studied phases of Zn_2_V_2_O_7_. The malleability can also be correlated with the value of Poisson's ratio. A material will be ductile if *ν* > 0.26, otherwise it will be brittle.^39^ The calculated values of Poisson's ratio, presented in Table 3, confirm the malleable character.

**Table 3:** Values of equilibrium volume *V*_0_ (Å^3^), bulk modulus *B*_H_ (GPa), shear modulus *G*_H_ (GPa), Young's modulus *Y* (GPa), Poisson's ratio *ν*, and Pugh's ratio *B*_H_/*G*_H_ for different phases of Zn_2_V_2_O_7_

Phases	*V*_0_	*B*_H_	*G*_H_	*Y*	*B*_H_/*G*_H_	*ν*	Ref.
α	598	88, 57 (ref. 21)	37	98	2.35	0.31	This work
β	283	130, 114 (ref. 21)	68	175	1.90	0.28	
γ	272	109, 71 (ref. 21)	38	103	2.85	0.34	
κ	515	112, 89 (ref. 21)	49	128	2.29	0.31	
δ	493	132, 127 (ref. 21)	54	142	2.46	0.32	

The mechanical performance of a material can also be signified by the number of useful indicators tabulated in Table 4. The machinability index μ^M^ (=*B*_H_/*C*_44_) is such an indicator that can be useful for describing the plasticity and lubricating behavior of a material.^40–42^ It is observed that a lower value of *C*_44_ gives better dry lubricity. However, a high value of *μ*^M^ exhibits excellent lubricating properties, high plastic strain value, lower friction value and feed forces. All phases in Zn_2_V_2_O_7_ suggest a high degree of machinability. Within the studied phases, β and δ are more machinable which is consistent with the high value of *B*_H_. The Kleinman parameter *ζ* (= (*C*_11_ + 8*C*_12_)/(7*C*_11_ + 2*C*_12_)) typically has a value between 0 and 1. According to the theory given by Kleinman,^43^ it is reasonable to consider that the bond bending contribution instead of bond stretching is dominant for the mechanical strength in almost all phases of Zn_2_V_2_O_7_ except the β phase due to its high value of *C*_11_. Indeed, this parameter reaches the maximum value when *C*_11_ equals *C*_12_. A perfect isotropic crystal has a zero value of the universal anisotropy factor *A*^U^.^44^ The degree of anisotropy is defined from the deviation of *A*^U^ = 0. In this case, all phases of Zn_2_V_2_O_7_ possess anisotropic nature. Understanding hardness is crucial for comprehending the elastic and flexible capabilities of a material. It is instructive to notice that the β-phase is reasonably harder than the other phases of Zn_2_V_2_O_7_, which is compatible with the calculated value of bulk modulus in Table 3.^45^

**Table 4:** Machinability index *μ*^M^, Kleinman parameter *ζ*, Vickers hardness *H*_V_ (GPa), density *ρ* (g cm^−3^), average wave velocity *v*_a_ (km s^−1^), and Debye temperature *Θ*_D_ (K) for the studied phases of Zn_2_V_2_O_7_

Phases	*μ* ^M^	*ζ*	*A* ^U^	*H*_V_	*ρ*	*ν*_a_	*Θ*_D_	Ref.
α	1.98	0.58	0.40	3.13	3.83	3.42	431	This work
β	4.33	0.27	−2.64	8.14	4.04	4.51	573	
γ	2.65	0.66	0.27	1.96	4.20	3.37	434	
κ	1.99	0.56	0.37	4.36	4.25	3.67	482	
δ	3.92	0.71	0.46	4.18	4.84	3.75	503	

Using the computed polycrystalline elastic moduli, the average elastic wave velocity *ν*_a_ is defined as follows:^46^2
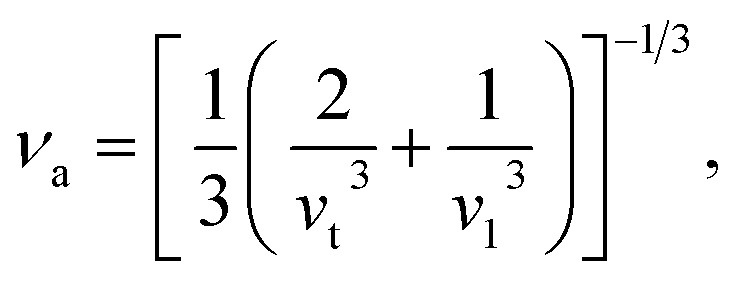
where *v*_t_ = (*G*/*ρ*)^1/2^ and *v*_l_ = ((*B* + 4*G*/3)/*ρ*)^1/2^ are the transverse and longitudinal wave velocities, respectively. Here, *ρ* defines the density of the material. The value of Debye temperature *Θ*_D_ was estimated using the following expression:^47^3
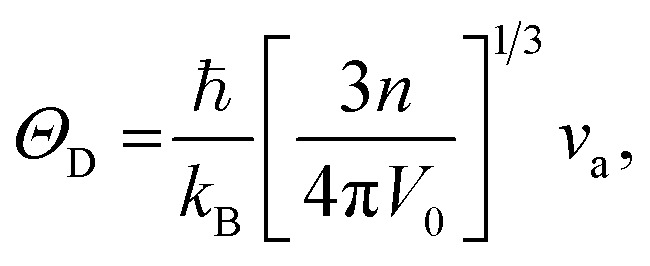
where ℏ is Planck's constant, *k*_B_ is Boltzmann's constant, *V*_0_ is the volume of the unit cell, *n* is the number of atoms and *ν*_a_ is the average wave velocity. The calculated values of average wave velocities along with the Debye temperatures for all of these phases are listed in Table 4. Referring to Table 4, the β-phase shows the highest elastic wave velocity compared to the other phases. A material with a high value of *Θ*_D_ signifies a higher melting temperature, greater hardness and stronger interatomic bonding strength. The β-phase is, in this case, harder identical with the hardness value presented in Table 4.

**Fig. 2:**
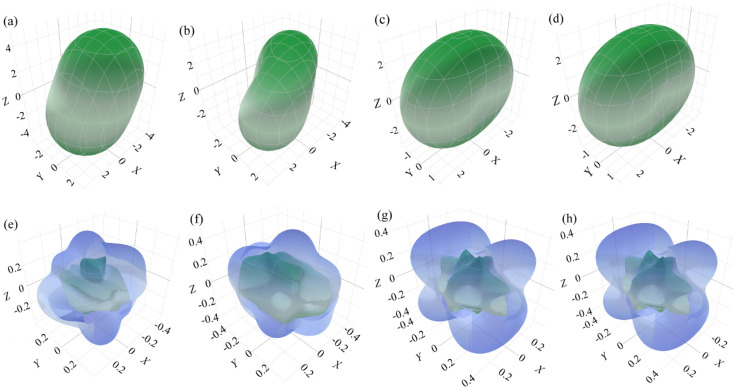
Three-dimensional plots of surfaces of the largest and smallest possible values of the linear compressibilities (top row) and Poisson's ratios (bottom row) for (a, e) α-, (b, f) γ-, (c, g) κ- and (d, h) δ-phases of Zn_2_V_2_O_7_, respectively.

In addition to the computed values regarding the elastic moduli, *e.g.*, bulk modulus/linear compressibility, Young's modulus, shear modulus and Poisson's ratio, it is possible to visualize the nature of anisotropy in three dimensions using ELATE, which is an open-source Python module for the analysis of elastic tensors.^27^ The first two moduli can be represented by a single unit vector and characterized by two angles *θ*(0 ≤ *θ* ≤ π) and *φ*(0 ≤ *φ* ≤ 2π) in spherical coordinates but the latter two are described by two orthogonal unit vectors, which can be parameterized by three angles *θ*, *φ* and *ξ*(0 ≤ *ξ* ≤ 2π). In the case of linear compressibility, most of the materials show positive compressibility indicating compression axially upon isostatic pressure. There may be some materials whose linear compressibility can be both positive and negative.^27,48,49^ For a negative linear compressibility, the structure expands in a particular direction upon pressure but the volume as a whole can be reduced. Although the moduli as a function of two unit vectors are difficult to represent in 3D, we determine Poisson's ratio (shear modulus not shown), following the work by Gaillac *et al.*,^27^ in the spherical coordinates (*θ*, *φ*) with the requirement of minimal and maximal values over all possible values of an extra dimension *ξ*. The 3-dimensional sketch of the largest and smallest possible values of the linear compressibility and Poisson's ratio of anisotropic surfaces for all of these studied phases except β is shown in Fig. 2. All the moduli expressed by either one unit vector or two orthogonal unit vectors as depicted in 3D diagrams are shown in Fig. 2, and it is observed that these 3D shapes strongly deviate from the spherical one, indicating a strong anisotropic nature compatible with the calculation of the universal anisotropy factor. The minimum and maximum values of different elastic moduli estimated by ELATE are tabulated in Table 5.

**Table 5:** Values of elastic moduli obtained from ELATE for the studied phases of Zn_2_V_2_O_7_

Phases	Young's modulus (GPa)		Linear compressibility (T Pa^−1^)		Shear modulus (GPa)		Poisson's ratio	
	Min	Max	Min	Max	Min	Max	Min	Max
α	71	128	2.9	5.1	27	50	0.11	0.51
γ	76	135	1.4	4.4	30	49	0.15	0.52
κ	86	158	1.6	5.1	36	62	0.18	0.57
δ	104	199	1.3	3.4	34	72	0.13	0.55

**Fig. 3:**
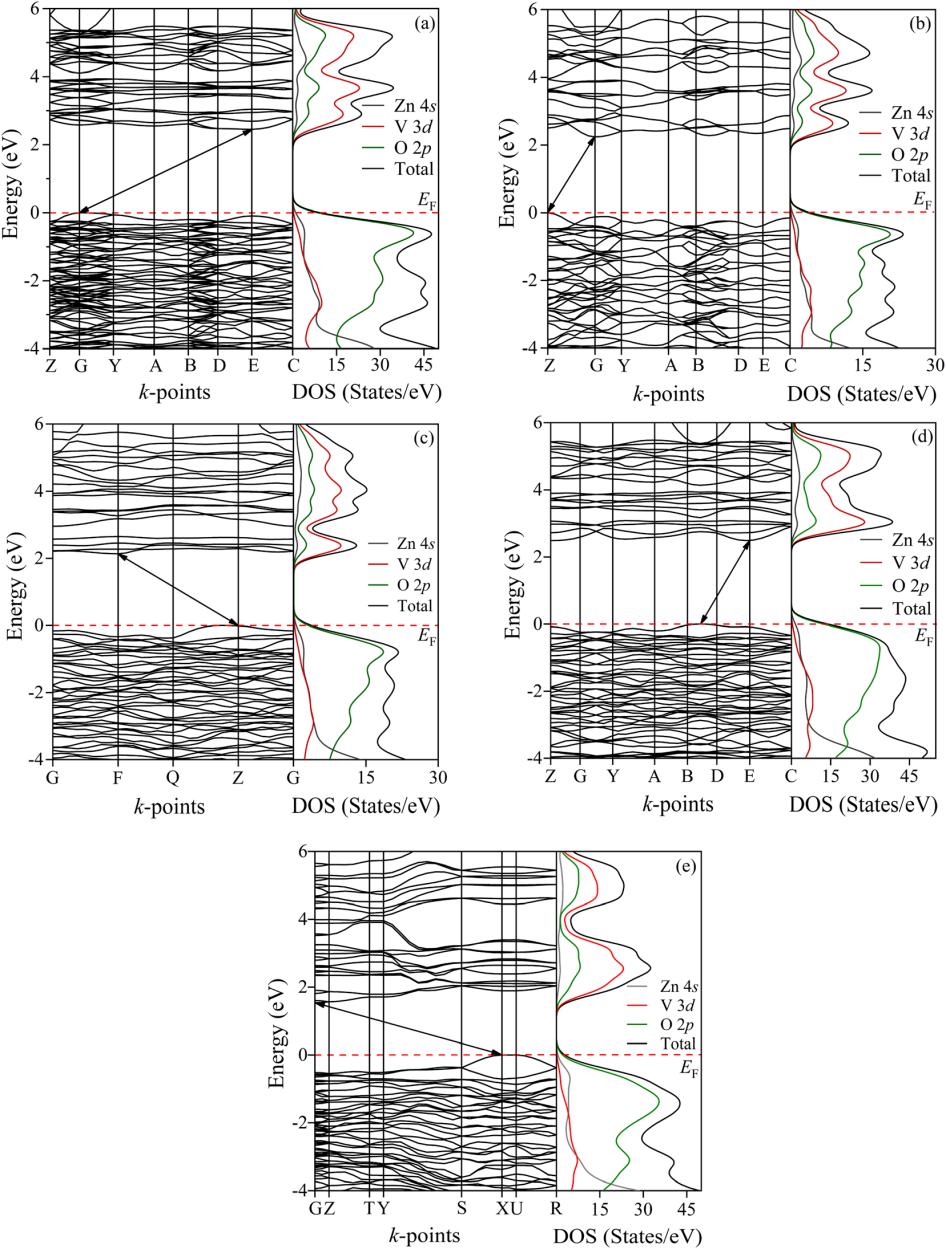
Energy dispersion curves for all the phases (a) α, (b) β, (c) γ, (d) κ and (e) δ of studied Zn_2_V_2_O_7_. The right panel shows the calculated density of states for the corresponding phases. The Fermi level *E*_F_ is adjusted to zero in the energy scale shown by the red dotted line.

To analyze the electronic behavior of zinc pyrovanadate, we first computed the energy dispersion and the corresponding density of states (DOS) of the studied phases under high-pressure situations using first-principles DFT calculations with the PBE functional. Fig. 3a shows the electronic band structure and the DOSs of an ambient α-phase of Zn_2_V_2_O_7_. It is shown that an ambient phase of studied pyrovanadate is an indirect band gap semiconductor, indicated by the solid black line arrows, with a wide gap of 2.45 eV listed in Table 6. The recent DFT calculation^21^-encoded CRYSTAL14 program using hybrid functionals, *e.g.*, B3LYP, HSE06, found the high value of energy band gap in comparison with the experimental value (∼3.5 eV) determined from the study of photoluminescence.^50^ The diffuse-reflectance spectra measurements^51,52^ reported a gap value 2.86 eV, which is almost consistent with the present calculations. Earlier estimation regarding the hetero-structured photocatalyst made by TiO_2_/vanadates presented a similar value of band gap energy of an ambient phase.^53^ Very recent computations^21^ using hybrid functionals have overestimated the band gap of vanadates inconsistent with the experimental value, but our calculations using the PBE functional are comparatively compatible with the experimental result given by diffuse-reflectance measurements.^51,54^

**Table 6:** Energy band gaps, density of states (DOS) and effective masses of these studied phases of Zn_2_V_2_O_7_ with the available theoretical and experimental values

Phases	Functionals	Gap	Energy gap (eV)	Energy gap (eV) (W–T theory)	DOS (states per eV)	Effective mass (*m*_0_)	
						Electron 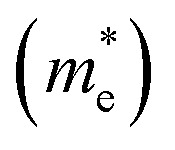	Hole 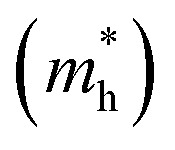
α	PBE	Indirect	2.45 (this work)	2.00	9.75	0.027	0.016
	PBE		2.29 (ref. 21)				
	HSE06		3.98 (ref. 21)				
	B3LYP		4.20 (ref. 21)				
	Expt.	—	2.5, 2.86 (ref. 51–53)				
			3.50 (ref. 50)				
β	PBE	Indirect	2.24 (this work)	1.75	4.33	0.008	0.035
	PBE		2.00 (ref. 21)				
	HSE06		3.63 (ref. 21)				
γ	PBE	Indirect	2.14 (this work)	1.65	3.72	0.015	0.022
	PBE	Direct	2.13 (ref. 21)				
	HSE06		3.78 (ref. 21)				
κ	PBE	Indirect	2.77 (this work)	2.10	14.10	0.527	0.014
	PBE		2.80 (ref. 21)				
	HSE06		4.56 (ref. 21)				
δ	PBE	Indirect	1.54 (this work)	1.49	3.19	0.032	0.052
	PBE		1.46 (ref. 21)				
	HSE06		2.91 (ref. 21)				

As can be presented in Fig. 3, the other phases of this pyrovanadate in our computations show the similar nature of semiconductivity, where the position of the top of the valence band (VB) and the bottom of the conduction band (CB) is different. According to the work given by Diaz-Anichtchenko *et al.*, the γ-phase exhibits a direct band gap semiconductor.^21^ The estimated values of the energy gap of β, γ, κ and δ-phases are 2.24, 2.14, 2.77 and 1.54 eV, respectively, as presented in Table 6. Moreover, the energy band gap of the post-γ phase (not shown) is nearly the same as the gap value of the γ-phase, so the prior prediction regarding the post-γ phase^21^ is not consistent yet. The right panel of Fig. 3 illustrates the calculated density of states (DOSs) for these bulk phases of Zn_2_V_2_O_7_ with atom projected contributions (partial DOS). It is seen that the top of the VB is mainly dominated by the O 2p orbitals, while the states near the bottom of the CB are dominated by V 3d orbitals with partial hybridization of O 2p orbitals. However, the contribution from Zn orbital electrons has no significant influence near the Fermi level so the energy band gap of these studied phases of pyrovanadate is almost comparable with the other metavanadates and orthovanadates.^21,55,56^

Out of these band gap energies for the phases of Zn_2_V_2_O_7_, the highest energy gap is seen in the κ-phase, which was a similar finding of the Díaz-Anichtchenko group under the DFT study.^21^ In their calculations, they have shown the variation of the energy gap under pressure. All phases of the pyrovanadate show a reduced trend as a function of pressure, resulting in an increase in the hybridization between V 3d and O 2p electrons similar to the metavanadate like ZnV_2_O_6_, except the κ-phase. However, the opposite trend of the band gap energy with pressure indicates the lack of attraction between bonding and antibonding states.^28^ Moreover, the pressure coefficient at ambient pressure in the κ-phase is a completely opposite sign and a much smaller value in comparison with the other phases. Such repulsion as well as the value of the pressure coefficient is responsible for showing the highest band gap energy in the κ-phase.

The effective masses for the electron 
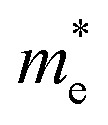
 or hole 
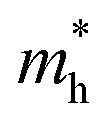
 at the band extremes can also be calculated from the energy dispersion using the following equation:4
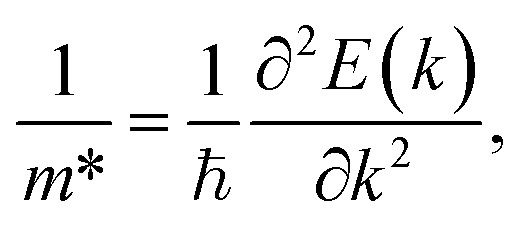
where ℏ is the reduced Planck constant and *E*(*k*) is the energy dispersion in terms of *k*-space. The effective masses were computed by fitting the parabolic equation of the energy in terms of the wave vector at the band extrema.^57^ The estimated values of the effective masses for the electron and hole for all the phases, expressed in units of free electronic mass *m*_0_, are listed in Table 6. Regarding the band structure result in the κ-phase compared with the other phases, the electron effective mass is much higher in the κ-phase as well as its DOS value. More DOS indicates more carrier density.^58^

**Fig. 4:**
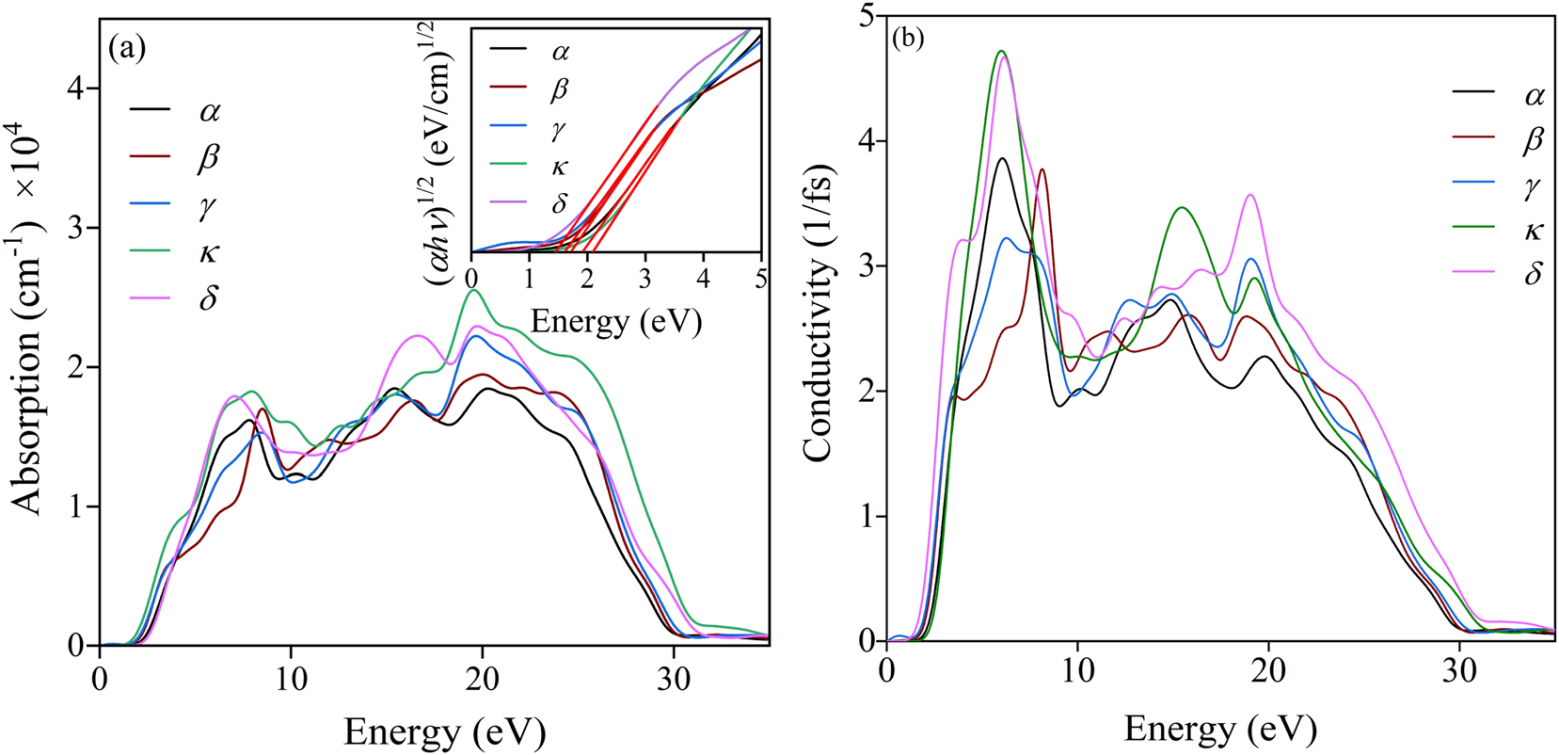
Energy dependence of (a) absorption spectra along with the Wood–Tauc plots (inset) and (b) conductivity for the studied phases of pyrovanadate.

The absorption coefficient is an important characteristic to understand the electronic nature of a material. The kind of electronic transition (either indirect or direct) in the energy bands can also be determined by studying the optical absorption spectra as a function of energy studied for the unpolarized light. The absorption spectra until 40 eV for the studied phases are depicted in Fig. 4a. It is seen that, the absorption for all these phases starts at some energy, which confirms the semiconducting behaviour already mentioned in the energy dispersion results. The absorption spectra imply that the light photon can efficiently be absorbed within the range of ∼7–25 eV, equivalent to the ultraviolet region; while the energy exceeds 25 eV, the absorption falls sharply.

The optical band gap energy (*E*_g_) can also be estimated by the Wood–Tauc (W–T) theory, as plotted in the insets of Fig. 4a, given by the relation *α*ℏ*ν* ∝ (ℏ*ν* − E_g_)^k^, where *α* is the absorbance, ℏ is the Planck constant, *ν* is the frequency and *k* is a constant associated with the different optical transitions.^55^ For an allowed indirect transition, the value of *k* is 2. By fitting with the best linear relation for a value of 2, the values of the band gap energy for different phases were also calculated, listed in Table 6. The spectra of photoconductivity, shown in Fig. 4b, start with some photon energy, which indicates that all the studied phases of Zn_2_V_2_O_7_ have a band gap as evident from the energy dispersion.

## Summary

We have investigated the pressure effects of zinc pyrovanadate using first-principles calculations under the framework of density functional theory (DFT). Within the pressure range below 10 GPa,^16^ the three phases and the other two predicted phases have been studied. All these phases are structurally consistent with the available experimental and theoretical results. All phases of the studied pyrovanadate are mechanically stable except β, elastically anisotropic as confirmed by the 3D results of elastic moduli done by ELATE and also malleable. The compressibility of Zn_2_V_2_O_7_ is surprisingly higher than that of the other *meta*- and *ortho*-vanadates. An indirect transition-type semiconductivity is exhibited in all of these phases *via* direct transition as mentioned in the γ-phase in the previous DFT work.^21^ The band gap energies as a function of pressure are decreased except κ-phase due to the smaller value of the pressure coefficient as well as the increase in repulsion between bonding and antibonding states. The post-γ-phase appearing at 10.8 GPa (ref. 16) was predicted to be the κ-phase, while the present calculations regarding post-γ-phase (not shown) do not find any similarity. Although the ordinary PBE functional in DFT slightly underestimates the energy band gap, the modified DFT approach, namely DFT + *U*_eff_, done by Dudarev *et al.*,^59^ could be expected to improve the description of electronic structures of the multiple vanadates, where the strong electronic interactions in V 3d orbital electrons would precisely be controlled.

## Conflicts of interest

There are no conflicts to declare.

## Supplementary Material
