# Correction: A computational study of the thortveitite structure of zinc pyrovanadate, Zn_2_V_2_O_7_, under pressure

**DOI:** 10.1039/d4ra90093f

**Published:** 2024-09-04

**Authors:** S. Reza, M. Maaza, M. S. Islam

**Affiliations:** a Department of Physics, University of Rajshahi Rajshahi 6205 Bangladesh sislamru@gmail.com; b UNESCO-UNISA Africa Chair in Nanosciences-Nanotechnology, College of Graduate Studies, University of South Africa Muckleneuk Ridge, PO Box 392 Pretoria South Africa; c Nanosciences African Network (NANOAFNET), Materials Research Dept., iThemba LABS-National Research Foundation of South Africa 1 Old Faure Road, Somerset West, PO Box 722 Western Cape 7129 South Africa

## Abstract

Correction for ‘A computational study of the thortveitite structure of zinc pyrovanadate, Zn_2_V_2_O_7_, under pressure’ by S. Reza *et al.*, *RSC Adv.*, 2023, **13**, 17212–17221, https://doi.org/10.1039/D3RA02426A.

The authors regret that there was an error in the first line in the introduction of the original article.

The text originally read, “The search for chemical systems suitable for lithium-ion batteries with good charge-exchange ability and high energy density^1–3^ has attracted the attention of researchers toward electrode materials to meet the energy demand in portable electronic devices such as cell phones, laptops, computers, digital cameras, and motor vehicles due to their storage capacity which commercial batteries made of graphite lack.” This sentence should read, “The search for chemical systems suitable for lithium-ion batteries with good charge-exchange ability and high energy density^1–3^ has attracted the attention of researchers toward electrode materials to meet the energy demand in portable electronic devices such as cell phones, laptops, computers, digital cameras, and motor vehicles due to their storage capacity instead of commercial batteries made of graphite.”

The authors regret that eqn (3) was shown incorrectly in the original article. The correct version of eqn (3) is as shown below. The corresponding section of the text in the manuscript should be adjusted according to this change, as detailed below.

The value of Debye temperature *Θ*_D_ was estimated using the following expression:^47^3
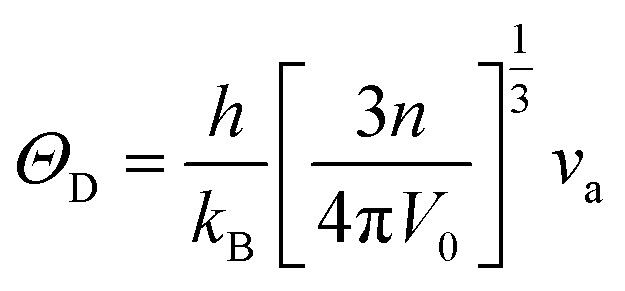
where *h* is Planck's constant, *k*_B_ is Boltzmann's constant, *V*_0_ is the volume of the unit cell, *n* is the number of atoms and *ν*_a_ is the average wave velocity.

The authors regret that eqn (4) was shown incorrectly in the original article. The correct version of eqn (4) is as shown below.4
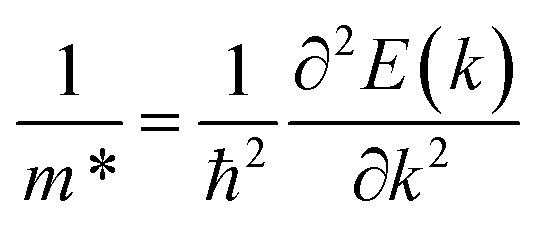


The Royal Society of Chemistry apologises for these errors and any consequent inconvenience to authors and readers.

